# Protein tyrosine phosphatases in skeletal development and diseases

**DOI:** 10.1038/s41413-021-00181-x

**Published:** 2022-01-28

**Authors:** Huiliang Yang, Lijun Wang, Christian Shigley, Wentian Yang

**Affiliations:** 1 Department of Orthopaedic Surgery and Orthopaedic Research Institute, West China Hospital of Sichuan University, Chengdu, 610041 Sichuan China; 2 Department of Orthopaedic Surgery, Rhode Island Hospital and Brown University Alpert Medical School, Providence, RI 02903 USA

**Keywords:** Bone, Calcium and phosphate metabolic disorders

## Abstract

Skeletal development and homeostasis in mammals are modulated by finely coordinated processes of migration, proliferation, differentiation, and death of skeletogenic cells originating from the mesoderm and neural crest. Numerous molecular mechanisms are involved in these regulatory processes, one of which is protein posttranslational modifications, particularly protein tyrosine phosphorylation (PYP). PYP occurs mainly through the action of protein tyrosine kinases (PTKs), modifying protein enzymatic activity, changing its cellular localization, and aiding in the assembly or disassembly of protein signaling complexes. Under physiological conditions, PYP is balanced by the coordinated action of PTKs and protein tyrosine phosphatases (PTPs). Dysregulation of PYP can cause genetic, metabolic, developmental, and oncogenic skeletal diseases. Although PYP is a reversible biochemical process, in contrast to PTKs, little is known about how this equilibrium is modulated by PTPs in the skeletal system. Whole-genome sequencing has revealed a large and diverse superfamily of PTP genes (over 100 members) in humans, which can be further divided into cysteine (Cys)-, aspartic acid (Asp)-, and histidine (His)-based PTPs. Here, we review current knowledge about the functions and regulatory mechanisms of 28 PTPs involved in skeletal development and diseases; 27 of them belong to class I and II Cys-based PTPs, and the other is an Asp-based PTP. Recent progress in analyzing animal models that harbor various mutations in these PTPs and future research directions are also discussed. Our literature review indicates that PTPs are as crucial as PTKs in supporting skeletal development and homeostasis.

## Introduction

Mammalian skeletal tissue is formed by two distinct mechanisms: intramembranous and endochondral ossification. The former produces many of the craniofacial bones directly from the condensation of mesenchymal progenitors; the latter is responsible for forming the rest of the skeleton by generating bone through a cartilage intermediate. Skeletal cells originate from three discrete embryonic lineages: (1) the cranial neural ectoderm, which gives rise to the jaw, teeth, auditory bones, and a portion of the craniofacial skeleton, (2) the paraxial mesoderm, which gives rise to the skull, vertebrae, sternum, and ribs of the axial skeleton, and (3) the lateral plate mesoderm, which gives rise to the limbs of the appendicular skeleton.^1^ During development, cells from these lineages migrate to sites of future cartilage and bone tissue to form dense aggregations, known as mesenchymal condensations, which dictate the size and shape of the skeletal element. At condensation sites, mesenchymal progenitors coordinate concerted expression of critical genes (e.g., transcription factors (TFs), growth factors, and matrix proteins) and activation of specific cellular signaling pathways to relay cellular messages and drive differentiation to form skeletal elements. Osteoclasts (OCs), derived from myeloid progenitors of hematopoietic origin, are specialized cells that absorb and remove the bone matrix and are essential for skeletal development. In adulthood, the integrity and homeostasis of the skeleton are maintained in equilibrium through the finely coordinated actions of osteoblasts (OBs), chondrocytes, and OCs.

Cells of the mesoderm and neural crest utilize a wide array of signaling pathways to proliferate, differentiate, and mature. Activation of these pathways upon the binding of growth factors, cytokines, and extracellular matrix (ECM) proteins to their cognate receptors results in signal transduction, at least in part, by activating specific protein tyrosine kinases (PTKs) that phosphorylate individual tyrosine residues of the signaling pathway components. Classical skeletal growth factors, such as fibroblast growth factors (FGFs),^2^ platelet-derived growth factors,^3^ vascular endothelial growth factors,^4^ and epidermal growth factors (EGFs),^5^ signal via receptor tyrosine kinases. Multichain cytokines and ECM proteins, such as IL6, collagens and laminin, signal by activating receptor or integrin-associated cytoplasmic Src family kinases (SFKs) and Janus family PTKs (JAKs).^6–8^ Moreover, chemokine signals utilize G protein-coupled receptors that activate SFKs and focal adhesion kinase (FAK).^9,10^ Given the large amount of literature available regarding the role of PTKs in the skeletal system, PTKs are beyond the scope of this review.

**Fig. 1:**
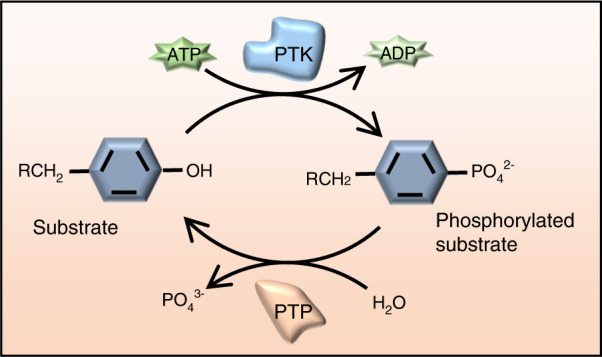
Dynamic regulation of PYP by protein tyrosine kinases (PTKs) and protein tyrosine phosphatases (PTPs). PTKs and PTPs catalyze transfer of phosphate groups between substrates. PTKs catalyze transfer of γ-phosphate from ATP to its substrates and PTPs remove the phosphate group from a phosphoprotein. PYP modulates various biological functions of many types of cells, including OCs, bone and cartilage cells, and is the most prevalent mechanism of protein regulation. Aberrant PYP due to altered expression or activity of PTKs or PTPs is associated with uncontrolled cell proliferation, differentiation, and diseases

Tyrosyl phosphorylation of signaling proteins in skeletal tissue, as in other tissues and cells, is tightly controlled at a steady-state level via concerted action of PTKs and protein tyrosine phosphatases (PTPs), a superfamily of proteins characterized by their enzymatic activity in removing the phosphate group from phosphorylated tyrosine residues (Fig. 1). Dysregulation of protein tyrosine phosphorylation (PYP) due to altered expression and/or activity of PTKs or PTPs can lead to skeletal development abnormalities, tumorigenesis, and degenerative diseases.^11–13^ In contrast to what is known about PTKs, less is known about PTPs in the skeletal system. Here, we review current knowledge about PTPs in the skeletal system, highlight recent discoveries, and propose new directions to advance this field of research.

## PTP classification

**Fig. 2:**
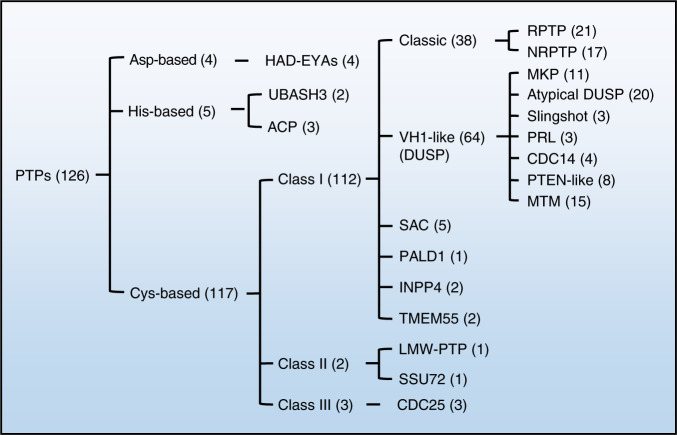
Classification of PTPs in the human genome. There are ~126 PTPs in the human genome. These PTPs are classified into three major groups based on their nucleophilic catalytic residue (Cys, Asp, or His) and topology. The numbers in parentheses indicate the members of PTPs included in each group. Only 9 PTPs are Asp- or His-based; the remaining 117 are Cys-based PTPs consisting of classes I, II, and III. Class I PTPs are further divided into six groups based on domain architecture and the degree of homology between catalytic domains. Classes II and III contain only two and three members, respectively. EYA eyes absent, UBASH3 ubiquitin-associated and SH3 domain-containing protein, ACP acid phosphatase, VH1 *Vaccinia* virus H1 gene product, DUSP dual-specificity phosphatase, RPTP receptor-like protein tyrosine phosphatase, NRPTP nonreceptor protein tyrosine phosphatase; MKP mitogen-activated protein kinase phosphatase, PRL phosphatase of regenerating liver, CDC cell division cycle, PTEN phosphatase and tensin homolog, MTM myotubularin, SAC sac phosphoinositide phosphatase, PALD1 phosphatase domain-containing paladin 1, INPP4 inositol polyphosphate-4-phosphatase, LMW low molecular weight, SSU72 RNA polymerase II subunit A C-terminal domain phosphatase SSU72

The skeletal system, similar to other tissues and cells, contains a wide variety of PTPs. Genome sequencing efforts have revealed at least 126 proteins with potential PTP activity in humans.^14–16^ To promote global collaboration and codify knowledge of PTPs, members of the PTP superfamily are grouped into three major types based on the nucleophilic catalytic residue (Cys, Asp, or His) in their catalytic motif and topology (Fig. 2).^14^ The conserved “signature motif (I/V)HCxxxxxR(S/T)” is shared by almost all Cys-based PTPs, including classes I, II, and III (Fig. 2).^14,16^

Cys-based class I PTPs are divided into six subclasses: classical phosphotyrosine (pTyr)-specific phosphatases, VH1-like/dual-specificity phosphatases (DUSPs),^17^ SAC phosphoinositide phosphatases, phosphatase domain-containing paladin 1 (PALD1), INPP4 phosphatases, and TMEM55 phosphatases (Fig. 2).^14–16,18^ Classical PTPs and DUSPs comprise ~91% of Cys-based class I PTPs. There are 38 genes encoding classical PTPs and 64 genes encoding DUSPs in humans. Classical PTPs are further divided into receptor-like PTPs (RPTPs) (21 members) and nonreceptor PTPs (NRPTPs) (17 members), both with high substrate specificity toward pTyr.^14,16^ In addition, DUSPs can be further divided into 7 groups: mitogen-activated protein kinase (MAPK) phosphatases (MKPs) (11 members), atypical DUSPs (20 members), slingshots (3 members), phosphatases of regenerating liver (PRLs) (3 members), cell division cycle 14s (CDC14s) (4 members), phosphatase and tensin homolog-like phosphatases (PTEN-like) (8 members), and myotubularin (MTM) (15 members) (Fig. 2).^14,16^

**Fig. 3:**
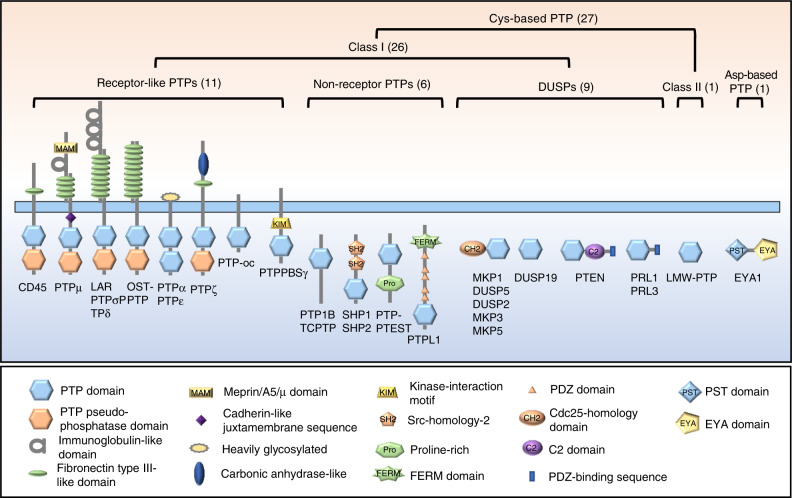
Schematic diagrams depicting the structures of 28 PTPs that are covered by this review and involved in skeletal development and diseases. Most PTPs belong to the Cys-based class I PTPs, which are further classified into receptor-like PTPs (RPTPs), nonreceptor PTPs (NRPTPs), and DUSP groups. Only one PTP (LMW-PTP) belongs to Class II; one PTP belongs to Asp-based PTPs. The diagrams were adapted from ref. ^15^

Class II consists of two members: low molecular-weight PTPs (LMW-PTPs) and SSU72. Class III PTPs include three mammalian CDC25 proteins that participate in cell cycle regulation (Fig. 2). Here, we focus on 27 Cys-based and 1 Asp-based PTPs known to be involved in skeletal development and diseases (Table 1; Fig. 3). Recent progress in our understanding of animal models with mutations in these genes are also discussed.

**Table 1:** Twenty-eight PTPs involved in skeletal development and human skeletal diseases

#	Gene/ID	Protein Names	Chromosome Location	Catalytic motif	Specificity	Skeletal development and diseases	Ref.
1	*Ptpra*/5786	RPTPα	20p13	HCSAGVGR	pTyr	OB	^41^
2	*Ptprc*/5788	CD45	1q31–q32	HCSAGVGR	pTyr	OC	^134,135^
3	*Ptprd*/5789	RPTPδ	9q23–p24.3	HCSAGVGR	pTyr	Ewing sarcoma, osteosarcoma,	^254–256^
4	*Ptpre*/5791	RPTPε, cyt-ptpε	10q26	HCSAGVGR	pTyr	OC	^137–139^
5	*Ptprf*/5792	LAR	1p34	HCSAGVGR	pTyr	OB, CC	^25,30^
6	*Ptprm*/5797	RPTPμ	18p11.2	HCSAGAGR	pTyr	OB	^41,46^
7	*Ptpro*/5800	PTP-oc	12p13–p12	HCSAGVGR	pTyr	OC	^142–150^
8	*Ptprr*/5801	PTP-SL, PTPPBSγ	12q15	HCSAGIGR	pTyr	CC	^214^
9	*Ptprs*/5802	RPTPσ	19p13.3	HCSAGVGR	pTyr	OB, CC	^25^
10	Ptprz/5803	RPTPζ	7q31.3	HCSAGVGR	pTyr	OB, CC, OA, osteosarcoma	^50,211–213,258^
11	*Ptprv*/148713	OST-PTP	1q32.1	HCSAGIGR	pTyr	OB, OC, CC	^53,55,56,58^
12	*Ptpn1*/5770	PTP1B	20q13.1–q13.2	HCSAGIGR	pTyr	OB, OC, CC	^41,58,68,215^
13	*Ptpn2*/5771	TC-PTP	18p11.3–p11.2	HCSAGIGR	pTyr	OC	^153^
14	*Ptpn6*/5777	PTP1C, SHP1	12p13	HCSAGIGR	pTyr	OB, OC	^70,72–75^
15	*Ptpn11*/5781	PTP1D, SHP2	12q24	HCSAGIGR	pTyr	OB, OC, CC, RA, NS, LS, metachondromatosis	^6,36–38,83,84,173–178,260,264^
16	*Ptpn12*/5782	PTP-PEST	7q11.23	HCSAGCGR	pTyr	OB, OC	^99,101,138,187^
17	*Ptpn13/5783*	PTPL1	4q21.3	HCSAGIGR	pTyr	Ewing sarcoma	^257^
18	*Dusp1*/1843	MKP1	5q34	HCQAGISR	PSer pThr, pTyr	OB, OC, CC	^103–106,193–196,225–227^
19	*Dusp2*/1844	PAC1	2q11	HCQAGISR	pSer, pThr, pTyr	OC	^203^
20	*Dusp5*/1847	hVH3	10q25	HCEAGISR	pSer, pThr, pTyr	OC	^200,201^
21	*Dusp6*/1848	MKP3	12q22–q23	HCLAGISR	pSer, pThr, pTyr	CC	^228^
22	*Dusp10*/11221	MKP5	1q41	HCQAGVSR	pSer, pThr, pTyr	CC, OA	^229,230^
23	*Dusp19*/142679	SKRP1	2q32.1	HCNAGVSR	pSer, pThr, pTyr	CC, OA	^232,233^
24	*Ptp4a1*/7803	PRL1	6q12	HCVAGLGR	pTyr	CC	^243^
25	*Ptp4a3*/606449	PRL3	8q24.3	HCVAGLGR	pSer, pThr, pTyr, PIP2	OC	^203^
26	*Pten*/5728	PTEN	10q23.3	HCKAGKGR	PIP3, pSer, pThr, pTyr	OB, OC, CC, OA, osteosarcoma	^114–116,205–208,234–238,259^
27	*Acp1*/52	LMW-PTP	2p25	VCLGNICR	pTyr	OB,	^128,129^
28	*Eya1*/2138	EYA1	8113.3	WDLDET	pSer, pTyr	OS	^14,251^

*OB* osteoblast, *OC* osteoclast, *CC* chondrocyte, *RA* rheumatoid arthritis, *NS* Noonan syndrome, *LS* Leopard syndrome, *OS* Otofaciocervical syndrome, *OA* osteoarthritis

## PTPs in osteoblast development, function, and bone homeostasis

OBs are morphologically cuboidal cells primarily responsible for the formation and maintenance of the vertebrate skeleton (osteogenesis).^19–21^ Similar to other cells, osteogenic differentiation of mesenchymal progenitors and the function of mature OBs are modulated by multiple intracellular signaling pathways that rely on tyrosyl phosphorylation. OBs originate from 2 distinct embryonic populations: the neural ectoderm and perichondral progenitors.^22^ Both give rise to immature OBs, also called OB precursors. Under internal and external stimuli, these precursors differentiate and become OBs. In addition, hypertrophic chondrocytes can directly transdifferentiate into OBs as an alternative source of osteogenic cells.^23,24^ OBs are postmitotic cells but are not terminally differentiated; they can further mature into osteocytes when surrounded by the bone matrix. PTPs not only modulate the fate determination of skeletal stem cells but also influence the proliferation, osteogenic differentiation, maturation of OB precursors, as well as the function of osteocytes. Below, we summarize 13 members of the PTP family known to be involved in OB development, functional regulation, and OB-related diseases.

### Classical RPTPs

RPTPs contain a single transmembrane domain and variable N-terminal extracellular domains that share homology with cell adhesion molecules. Most RPTPs contain two tandem PTP domains in their intracellular regions. The membrane-proximal PTP domain is usually responsible for most catalytic activity, whereas the distal PTP domain has weak, if any, catalytic activity.^15^ Collectively, the structural characteristics of RPTPs enable direct coupling of extracellular adhesion-mediated events to the regulation of intracellular signaling pathways in skeletal cells.

Leukocyte common antigen-related RPTPs (LAR family RPTPs) comprise three members: RPTPδ (*Ptprd*), RPTPσ (*Ptprs*), and LAR (*Ptprf*). Each member has a cytoplasmic region with two tandem phosphatase domains and an extracellular region with fibronectin type III-like (FN-III) and immunoglobin-like domains (Fig. 3).^15,25^ LAR family RPTPs regulate several critical development events by negatively influencing growth factor receptor signaling, such as EGFR, Met/hepatocyte growth factor receptor (HGFR), and RET.^26,27^ Additionally, LAR RPTPs are reported to positively modulate canonical Wnt/β-catenin signaling. Mice lacking both RPTPσ and LAR exhibit mandibular and maxillary bone and cartilage patterning defects, developing micrognathia, cleft palate, and macroglossia.^25^ The phenotype strongly resembles Pierre Robin Sequence (PRS) in humans.^28^ Mechanistically, LAR deficiency causes elevated BMP-SMAD signaling and represses canonical Wnt signaling in mouse embryonic tissues.^25^ These findings suggest that LAR RPTPs function as pivotal regulators of craniofacial morphogenesis, providing insight into the etiology of PRS.

**Fig. 4:**
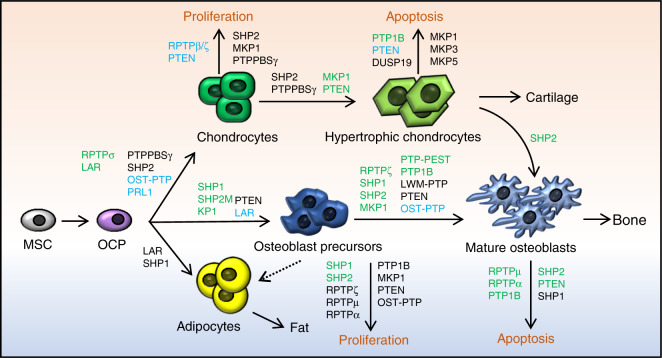
PTPs regulate skeletogenesis by influencing the proliferation and fate determination of skeletal stem cells, the differentiation and maturation of chondroid, adipose, and osteoblastic cell lineages, and the function of osteoblasts and chondrocytes. Developmental trajectories of OBs, chondrocytes, and adipocytes are connected by arrows; regulatory PTPs are involved, and their biological function in these processes is indicated. Green, black, and blue represent PTPs that play a positive, negative, and conflicting or unclear regulatory role, respectively

**Fig. 5:**
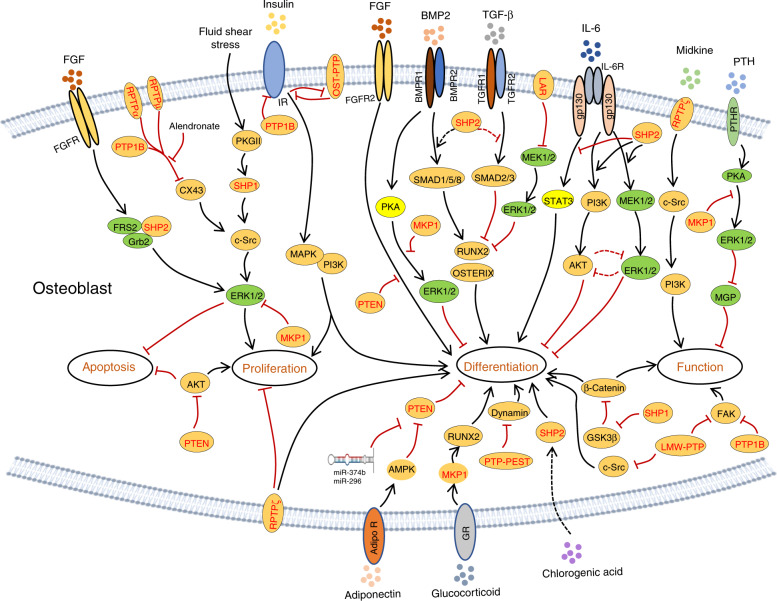
PTPs modify multiple signaling pathways that differentially regulate the viability, proliferation, differentiation, and function of osteoblastic cells. Established signaling pathways in OBs are connected by lines with arrows indicating “promotion” and “┴” indicating inhibition. PTPs involved in each signaling pathway are marked in red

LAR also negatively influences the adipogenic fate of mesenchymal stem cells (MSCs). Knockdown or overexpression of LAR promotes or suppresses adipogenic differentiation, respectively, in both 3T3-L1 preadipocytes and MSCs. Such negative regulation is likely mediated by LAR’s regulation of insulin receptor (IR) phosphorylation and signaling (Fig. 4).^29^ Consistent with previous findings, LAR overexpression was found to decrease ERK activation but promote osteogenic differentiation of MC3T3-E1 preosteoblasts, as evidenced by increases in *Alp, Ibsp, Dlx5*, *Bglap*, and *Runx2* transcript abundance (Fig. 5).^30^

However, these claims were challenged by other independent genetic and biochemical studies. For example, deletion of MEK1/2^31^ and ERK1/2^32,33^ in osteoprogenitors results in severe osteopenia, limb deformity, and defective mineralization. This phenotype is strikingly similar to that of cleidocranial dysplasia seen in humans and mice, which is associated with the absence of functional RUNX2^34,35^ and SHP2 in osteochondroprogenitors (OCPs)^36,37^ and OBs.^38^ Collectively, LAR deletion promotes adipogenic differentiation of MSCs. However, its role in regulating ERK activation and OB differentiation needs to be further investigated to rule out if LAR overexpression has off-target effects. A genetic rescue experiment would be helpful for resolving the discrepancy observed in vivo and in vitro.

RPTPα (encoded by *Ptprα*) is ubiquitously expressed, and its enzymatic activity is regulated by tyrosine and serine phosphorylation.^39,40^ Lezcano et al. reported that RPTPα is involved in the survival and proliferation of OBs treated with the bisphosphonate family drug alendronate (ALN) (Figs. 4, 5).^41–43^ Importantly, RPTPα activity is inhibited by ALN in ROS 17/2.8 OBs.^41^ Therefore, RPTPα may serve as a substrate of bisphosphonates in OBs to prevent apoptosis and promote cell proliferation, though the molecular mechanism remains elusive.

RPTPμ (encoded by *Ptprm*) is predominantly expressed in neuronal cells, the lung epithelium, MSCs, and endothelial and cardiac muscle cells.^15,44^ Early studies have shown that RPTPμ levels are directly proportional to adipogenic differentiation in 3T3-L1 preadipocytes and MSCs. This regulation is mediated by RPTPμ dephosphorylation of p120 catenin and reduced cytoplasmic accumulation.^45^ Moreover, RPTPμ is reportedly expressed in osteocytes; its deletion causes a significant reduction in cortical bone, but without an apparent effect on trabecular bone mass.^46^ Mechanistically, RPTPµ may regulate mechanosignaling in osteocytes.^46^ In other studies, RPTPμ was shown to associate with connexin (Cx) 43 hemichannels. Interruption of this interaction by ALN promotes OB survival and proliferation (Figs. 4, 5).^41^ Based on these lines of evidence, it is concluded that RPTPμ negatively regulates OB survival and proliferation.

RPTPζ (encoded by *Ptprz*) contains an N-terminal carbonic anhydrase-like domain, an FN-III, and a large intervening sequence (Fig. 3).^15,47^ RPTPζ can bind to cell adhesion molecules, growth factors (midkine, pleiotrophin, FGF2), and ECM molecules (tenascin-C, tenascin-R, amphoterin),^48,49^ and it is only detectable in fully differentiated OBs. Mice lacking RPTPζ are defective in OB maturation, as revealed by a reduction in *Bglap* and *Ibsp* in calvarial OBs and a decreased new bone formation rate, resulting in osteopenia.^50^ Therefore, by promoting OB terminal differentiation but repressing its proliferation, RPTPζ is required for osteogenesis (Fig. 4). Mechanistically, ligand binding to RPTPζ stimulates its enzymatic activity, which in turn activates c-Src, PI3 kinase (PI3K), and MAPK (Fig. 5).^51^ However, Meng et al. reported that binding of pleiotrophin to RPTPζ suppresses its catalytic activity in glioblastoma cells both in vitro and in vivo, causing enhanced tyrosyl phosphorylation of β-catenin and cell adhesion.^52^ Since this signaling machinery also exists in osteoblastic cells, future studies should investigate whether RPTPζ influences OB development and function via β-catenin.

Osteotesticular PTP (OST-PTP; encoded by *Ptprv*) is an RPTP primarily expressed in mouse OBs and gonads.^53,54^ An NCBI database search revealed that *Ptprv* is a pseudogene, though its homolog with PTP activity in humans has not yet been identified. OST-PTP mRNA is upregulated following OB differentiation, with predominant expression in differentiated and early mineralizing OBs.^53,55^ Administration of OST-PTP-specific antisense oligonucleotides to primary OBs reduces their differentiation into mature OBs in vitro;^53^ such developmental stage-specific expression of OST-PTP was also demonstrated by Dacquin et al.^54^ Moreover, OST-PTP expression can be modulated in response to known OB regulators, including parathyroid hormone (PTH) and vitamin D_3._^56^ Given the similar spatiotemporal expression patterns of OST-PTP and RUNX2, a potential relationship between these two molecules during skeletogenesis has been proposed.^56^ Thus, OST-PTP appears to be required for OB differentiation from immature OB precursors to mature OBs but not OB proliferation. Interestingly, Ferron et al. reported that OST-PTP negatively regulates OB proliferation and differentiation stimulated by insulin^57,58^ by dephosphorylating phosphorylated Tyr1150/1151 of IR.^58^ In addition, OST-PTP inhibition is able to enhance insulin signaling and OB formation (Fig. 5).^57,58^ In contrast, insulin signaling inhibits OST-PTP and stimulates OB differentiation by promoting the production of undercarboxylated osteocalcin,^57^ which, in turn, increases insulin sensitivity and induces more insulin production. Mutual regulation of OST-PTP and IR perfectly explains the hypoglycemic, obese, and glucose intolerance phenotypes of *Ptprv*^*−/−*^ mice.^59^ In summary, OST-PTP negatively regulates OB proliferation, but its role in OB differentiation remains unclear (Fig. 4).

### Classical NRPTPs

NRPTPs are primarily localized in a variety of intracellular compartments, including the cytosol, plasma membrane, and endoplasmic reticulum (ER). Each NRPTP contains a single catalytic domain connected to variable sequences that modulate its activity and intracellular localization.

PTP1B (encoded by *Ptpn1*) was the first PTP discovered; it is widely expressed and predominantly localized in the ER.^60^ General information regarding the structure and function of PTP1B is available in multiple reviews.^15,57,61,62^ Although *Ptpn1*^*−/−*^ mice develop normally, they display defects in glucose and insulin tolerance.^63,64^ Recently, IR and IR substrate 1 (IRS-1) were shown to be substrates for PTP1B.^57,65–67^ Inhibition of PTP1B by titanium^68^ or Cx43-associated PTP1B by bisphosphate^41^ promotes pre-OB adhesion by sustaining phosphorylation of FAK Tyr397 or by increasing OB proliferation and resisting apoptosis, respectively (Figs. 4, 5).

SHP1 and SHP2 (encoded by *Ptpn6* and *Ptpn11*, respectively) belong to the Src homology 2 domain-containing cytoplasmic PTP family. SHP1 is predominantly expressed in hematopoietic cells; SHP2 is ubiquitously expressed at variable levels in different tissues.^69^ SHP1 and SHP2 may have “positive” (promoting) or “negative” (inhibiting) signaling roles depending on the cell type. The motheaten (*me/me*) and viable motheaten (*mev/mev*) mutations that lead to SHP1 deletion (*Ptpn6*^*me/me*^) or its enzymatic activity reduction (*Ptpn6*^*mev/mev*^) cause autoimmune diseases, manifesting as a *“*motheaten*”* appearance of the skin.^70,71^ Affected *Ptpn6*^*mev/mev*^ mutants display osteoporotic pathology, which was recently reported to be due to the fate switch of mesenchymal progenitors favoring adipogenesis rather than osteogenesis (Fig. 4).^72^ Mechanistic studies show that SHP1 regulates MSC differentiation by influencing lineage-specific TF expression (e.g., C/EBPs, PPARγ, and Runx2).^72,73^ SHP1 deletion elevates glycogen synthase kinase-3β (GSK3β) activity and subsequent β-catenin degradation. In turn, β-catenin degradation leads to impaired OB differentiation and matrix mineralization, partially contributing to the osteoporotic phenotype of *Ptpn6*^*mev/mev*^ mutants (Fig. 5).^72,73^ SHP1 is also expressed in cells of the osteoclastic lineage. SHP1 deficiency or hypomorphic mutations enhance osteoclastogenesis, OC resorptive activity, and ultimately, bone mineral loss.^74,75^ These findings, however, conflict as to whether the osteoporotic phenotype of *Ptpn6*^*mev/mev*^ mice results from aberrant OB or OC differentiation.^72,73^ Conceivably, mouse models in which SHP1 is deleted in an OB- or OC-specific manner might be an ideal design to address these questions.

Mechanical loading induces anabolic responses of OBs and regulates bone quality and mineral homeostasis. Fluid shear stress activates c-Src and SHP1, which promote OB proliferation and survival in vitro (Figs. 4, 5).^76^ Several lines of evidence suggest that c-Src activation in OBs is mediated by dephosphorylation of inhibitory pY529 (in humans, pY527) through SHP1 and SHP2.^76,77^ One question remains: how are SHP1 and SHP2 activated and recruited to c-Src for their action in osteoblastic cells.

In contrast to SHP1, SHP2 null mutation causes early embryonic lethality.^78,79^ Somatic SHP2 mutations are associated with several human diseases that have skeletal manifestations. *Ptpn11* mutations lead to increased SHP2 enzymatic activity and altered activation of the Ras/RAF/ERK signaling cascade responsible for Noonan syndrome (NS). NS is an autosomal dominant disorder characterized by dysmorphic facial features, proportionate short stature, and decreased bone mineral density (BMD),^80,81^ as well as heart disease.^82,83^ To circumvent the lethality of SHP2 null mutations and study SHP2’s function in the osteoblastic cell lineage, *Ptpn11* floxed alleles have been created^13,77^ and bred with a series of Cre alleles to target osteoblastic cells at various developmental stages. Deletion of SHP2 in the murine limb bud mesenchyme via *Prrx1*-*Cre*-mediated excision of *Ptpn11* floxed alleles causes dwarfism, limb and chest deformities, and defective mineralization in the skull.^37^ These skeletal abnormalities are associated with impaired OB maturation and massive chondrocyte formation, as well as compromised ERK and AKT activation (Figs. 4, 5).^37^ Parallel studies performed by Zuo et al. showed that SHP2 indirectly regulates SOX9 phosphorylation at the AGC family kinase consensus motif “R/KxxST”, which is mediated by protein kinase A (PKA) in *Prrx1*^*+*^ progenitors and their derivatives.^36^ Enhanced SOX9 phosphorylation increases its protein stability, sumoylation, transcriptional activation, and subsequent chondrogenic gene expression.^36^ SHP2 deletion in *Prrx1*^*+*^ mesenchymal progenitors also enhances TGFβ- and suppresses BMP2-evoked signaling, leading to defective OB differentiation (Fig. 5).^84^ Importantly, somatic SHP2 deletion in *Prrx1*^*+*^ mesenchymal progenitors causes neoplastic cell growth and cartilage tumor formation, suggesting that SHP2 loss-of-heterozygosity mutation is a molecular mechanism of cartilage tumor formation.^36^ In addition to its indispensable role in regulating the fate of MSCs, SHP2 is required for the maturation and function of OBs and osteocytes. Mice lacking SHP2 in *Bglap*^*+*^ mature OBs exhibit decreased BMD, impaired osteocyte canalicular network formation, and eventually skeletal degeneration. At the molecular level, SHP2 deletion was found to substantially decrease expression of *Osx* and OSTERIX, suggesting that SHP2 influences OB and osteocyte maturation, at least by controlling OSTERIX expression and transcriptional activity.^38^

SHP2 modulates IL6 signaling and the course of rheumatoid arthritis. The IL6 signaling pathway has little impact on OB proliferation but negatively influences OB differentiation.^6^ This action is mediated through activation of SHP2, ERK, and AKT, as treatment of MC3T3 cells with small molecule inhibitors of SHP2, ERK, and AKT (PHPS1, UO126, and LY294002, respectively) restored expression of osteogenic genes and deposition of calcium in vitro (Fig. 5).^6^ Nonetheless, these findings have been challenged by recent genetic studies in which ablation of SHP2,^36–38,84^ ERK1/2,^31,32,85^ and AKT^86,87^ in the osteoblastic cell lineage halted their osteogenic differentiation and maturation. IL6 signals through gp130, which also activates signal transducer and activator of transcription 3 (STAT3) via JAK family kinases, to promote osteogenic differentiation (Fig. 5).^88,89^ SHP2 reportedly negatively modulates IL6-evoked STAT3 phosphorylation and activation upon binding to gp130Y759.^90,91^ Conceivably, SHP2 might influence OB differentiation and function by modifying multiple signaling pathways, including the ERK, AKT, and STAT3 pathways.

SHP2 also modulates FGF-evoked GRB2/FRS2 signaling complex formation and ERK activation, which are critical for skeletal development and homeostasis.^19,92^ An early study using chimeric embryos showed that SHP2 is essential for the outgrowth of limb buds and that *Ptpn11*^*−/−*^ embryonic stem cells fail to contribute to the mesenchyme of the progress zone. This failure is phenotypically reminiscent of *Fgfr1* mutant chimeric embryos, strongly suggesting that SHP2 is required for FGFR1 signaling to properly guide limb bud development.^93^ In contrast, FGFR2 gain-of-function (GOF) mutations C342Y (Crouzon syndrome) or S252W (Apert syndrome) inhibit OB differentiation and dramatically induce apoptosis.^94^ Furthermore, FGF2 overexpression leads to increased apoptosis in the mouse calvaria, suggesting that FGF acts as a cell death inducer with distinct effects on proliferating and differentiating OBs (Fig. 5).^94,95^ Moreover, SHP2 overexpression in bone marrow-derived MSCs appears to drive osteogenic differentiation both in vitro and in vivo.^96^ Although a lentiviral bicistronic vector was used to express SHP2 and a green fluorescent protein reporter, the variable levels of SHP2 in lentiviral-infected MSCs across different timepoints suggest that changes in the osteogenic gene profile might not be related to SHP2.^96^ Moreover, SHP2 is involved in the proliferation of MSCs and their osteoblastic differentiation induced by chlorogenic acid via the PI3K/AKT pathway (Fig. 5).^97^

PTP-proline-, glutamate-, serine-, and threonine-rich sequences (PTP-PEST, encoded by *Ptpn12*) are present in classical NRPTPs.^98–100^ Eleniste et al. reported that PTP-PEST regulates OB differentiation and migration by modifying the phosphorylation and GTPase activity of dynamin (Figs. 4, 5).^101^ Immunoprecipitation assays have revealed that PTP-PEST and dynamin form protein complexes in OBs. PTP-PEST inhibition increases and PTP-PEST overexpression decreases phosphorylation of dynamin in OBs. Importantly, the phosphorylation status of dynamin is associated with its GTPase activity. Moreover, PTP-PEST overexpression reverses the c-Src-mediated phosphorylation and GTPase activity of dynamin.^101^ Collectively, PTP-PEST positively regulates OB differentiation but inhibits OB migration by influencing dynamin phosphorylation, and dynamin phosphorylation is modified by both c-Src and PTP-PEST. Given that PTP-PEST also regulates c-Src activation, it remains unclear whether PTP-PEST modifies dynamin phosphorylation directly or indirectly via activation of c-Src.

### DUSPs

DUSPs are a heterogeneous group of protein phosphatases that dephosphorylate both pTyr and phosphoserine (pSer)/phosphothreonine (pThr) residues on the same substrate. DUSPs have been implicated as critical modulators in skeletal development and diseases. DUSPs are divided into seven subgroups based on sequence similarity, including MKPs, slingshots, PRLs, CDC14s, PTEN-like, MTMs, and atypical DUSPs. Herein, we only focus on MKP1 and PTEN because the function of the remaining DUSPs in the skeletal system is unknown.

The MAPK signaling pathway is indispensable for skeletal development and homeostasis.^31,102^ MKPs are a family of DUSPs that negatively regulate MAPK activation and their downstream signaling events in various types of cells. MKPs dephosphorylate conserved threonine and tyrosine residues within the activation loop of ERKs, c-Jun NH2 terminal kinase (JNK), and p38. MKP1 KO mice display increased OB proliferation but impaired maturation and function, resulting in overall reduced bone mass.^103^ Such studies have also demonstrated that MKP1 inhibits OB proliferation by negatively regulating cyclin D1 expression by dephosphorylating pERK1/2 (Fig. 5).^104,105^ Calvarial OBs from MKP1 mutants exhibit reduced expression of *Bglap*, *Runx2*, and *Alp* and an attenuated PTH response in vitro.^104,105^ Further investigation revealed that PTH inhibition of OB mineralization is MKP1 dependent through p38 regulation in early OBs and ERK1/2 in mature osteoblastic cells (Fig. 5).^105^ MKP1 has also been reported to promote BMP2-evoked osteogenic differentiation of C2C12 cells by dephosphorylating pERK1/2^106^ and dexamethasone-induced osteoblastic differentiation of dermal fibroblasts by dephosphorylating RUNX2 pSer125 (negative regulatory residue).^107^ Collectively, MKP1 appears to negatively regulate OB proliferation, though it is required for osteogenic differentiation (Fig. 4) and OB anabolic responses to PTH, BMP2, and glucocorticoids (Fig. 5).^106–108^

PTEN (encoded by *Pten)* is a DUSP that counters the activity of PI3K.^109,110^ Inactivation of PTEN in humans and mice has established PTEN as a bona fide tumor suppressor.^111,112^ At the molecular level, PTEN is primarily responsible for dephosphorylation of the lipid second messenger PtdIns(3,4,5)P3 to counterbalance PI3K upon various stimuli.^113,114^ Cells of the osteoblastic lineage express both PI3K and PTEN. To investigate the role of PTEN in bone cells, several Cre transgenes have been used to ablate PTEN in murine osteoblastic cells at various developmental stages.^114,115^ Dermo-1 belongs to the basic helix-loop-helix TF family and is expressed in limb buds at Day 10.5 post coitum, becoming restricted to the perichondrium in adulthood. Mice lacking PTEN in undifferentiated *Dermo1*^*+*^ mesenchymal cells show increased cell proliferation and osteogenic differentiation and expanded bone matrix as a result of augmented FGF but repressed SPRY2 signaling.^115^ Affected mutants develop short, wide, tubular bones, which can be partially rescued by deletion of FGFR2, suggesting that FGF signaling is the major mediator of *Pten* deletion in osteoprogenitors (Fig. 5).^115^ PTEN deletion in *Col2α1*^+^ osteochondroprogenitors leads to increased skeletal size, trabecular volume, and cortical bone thickness. Mutant chondrocytes show elevated levels of AKT and S6 phosphorylation, indicative of increased mammalian target of rapamycin (mTOR) and 3-phosphoinositide-dependent protein kinase 1 activity. Interestingly, cell proliferation in growth plate cartilage is comparable between controls and mutants, suggesting that PI3K/AKT/S6K signaling primarily regulates cell size, rather than cell proliferation, in this setting.^116^ Importantly, mice with PTEN deletion in mature OBs have a normal skeletal size, but their BMD increases progressively throughout life.^114^ These mutants also show improved endochondral bone formation and fracture healing.^117,118^ Taken together, PTEN negatively regulates osteogenic differentiation in MSCs and OCPs and the proliferation and maturation of OBs (Fig. 4).

The adiponectin signaling pathway is reported to negatively regulate PTEN expression and promote osteogenic differentiation of MSCs (Fig. 5).^119^ Consistent with these findings, inhibiting PTEN expression using miR-374b and miR-296 facilitates osteoblastic differentiation of MSCs (Fig. 5).^120,121^ Moreover, PTEN plays a crucial role in craniofacial development. Inactivation of PTEN in neural crest cells leads to craniofacial malformation due to altered cell proliferation and differentiation.^122^ Mechanistically, PTEN abundance is modulated by the NUMB endocytic adaptor protein via the posttranslational ubiquitin–proteasome pathway in osteoblastic cells.^123^

### Cys-based class II PTPs

LMW-PTP (encoded by *Acp1*) is expressed in all organisms, whereas most other PTPs are expressed exclusively in eukaryotes.^16,124^ LMW-PTP enzymatic activity is regulated by phosphorylation at Tyr131 and Tyr132.^125,126^ Zambuzzi et al. reported that OB differentiation requires c-Src activation, which is negatively regulated by LMW-PTP via dephosphorylation of the activation site c-Src Tyr416 (Figs. 4, 5).^127,128^ LMW-PTP also regulates OB adhesion and spreading by modulating FAK activation through Y397 dephosphorylation.^129,130^ Therefore, LWM-PTP functions as a negative regulator to modulate both c-Src and FAK activation during OB differentiation, adhesion, and spreading (Fig. 5).

## PTPs in osteoclastogenesis and osteoclast functional regulation

**Fig. 6:**
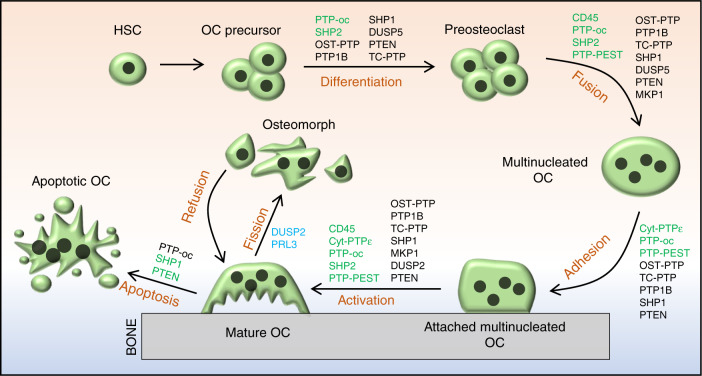
Schematic diagrams depicting the classical osteoclastogenic processes and novel osteoclast fission in which PTPs are implicated as playing a regulatory role. PTPs denoted in green or black indicate that they positively or negatively, respectively, modulate the commitment and differentiation of HSCs to myeloid cells, the fusion of OC precursors, and the adhesion, activation, and apoptosis of OCs. DUSP2 and PRL3 are reported to be highly expressed in osteomorphs, suggesting that they play a role in the fission of OCs. Their specific mechanism and function remain unclear, and they are denoted in blue

**Fig. 7:**
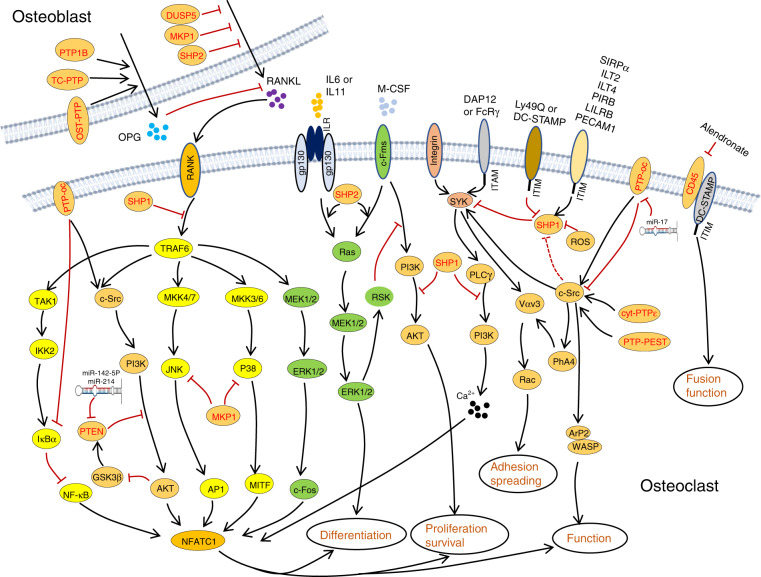
PTPs regulate osteoclastogenesis by directly controlling multiple critical signaling pathways in osteoclasts and indirectly regulating synthesis and secretion of osteoclastic cytokines by OBs. Established signaling pathways are connected by lines with arrows or “┴” to indicate a promoting or inhibiting role during osteoclastogenesis. Involved PTPs are denoted in red

OCs are giant multinucleated cells that differentiate from pluripotent hematopoietic stem cells (Fig. 6).^131^ OCs are present on bone surfaces and are primarily responsible for resorption of ECM proteins and minerals of the skeleton under various physiological and pathological conditions. Macrophage colony-stimulating factor (M-CSF) and receptor activator of nuclear factor kappa-B ligand (RANKL)-evoked cellular signaling are essential for osteoclastogenesis (Fig. 7).^131,132^ Similar to OBs, PYP is pivotal in regulating OC development and function, and dysregulation of PYP in OCs leads to osteopetrosis, osteoporosis, osteolysis, and bone metastasis of soft tissue cancers.^132^ Below, we review 14 PTPs involved in osteoclastogenesis and OC-related skeletal diseases in humans and mice.

### Classical RPTPs

CD45 (encoded by *Ptprc*) is known as a common leukocyte antigen, and RPTP is present in all cells of the hematopoietic lineage.^133^ CD45 dephosphorylates both the activation and inhibitory sites of SFKs to serve as a dual regulator in a cellular context-dependent manner.^134^ Mice lacking CD45 are osteopetrotic due to impaired OC fusion and function.^134^ CD45-deficient OCs exhibit abnormal morphology, a reduced number of nuclei, decreased secretion of metalloproteinase 9 (MMP9) and MT1-MMP as a consequence of an aberrant response to RANKL, increased Src activation, and decreased dendritic cell-specific transmembrane protein (DC-STAMP) expression.^134^ Additionally, CD45 inhibition by ALN reduces multinucleated OC formation and impairs OC bone resorptive activity.^135^ Overall, CD45 is required for OC development and function (Fig. 6).

Cyt-PTPε (encoded by *Ptpre*) is a member of the PTPε family, which also includes RPTPε, p67 PTPε, and p65 PTPε. Cyt-PTPε is selectively expressed in OCs (Fig. 3).^26,136,137^ PTPε KO mice are osteopetrotic due to impaired OC function.^137,138^ Mechanistically, cyt-PTPε acts as a feedback regulator and maximizes the activity of integrin-associated c-Src by modulating the structure and dynamics of podosomes (Fig. 7).^139^ Thus, cyt-PTPε might serve as a therapeutic target for diseases caused by overactivation of OCs, such as osteoporosis. To this end, substantial efforts have been made to develop specific inhibitors of cyt-PTPε, some of which are very promising. For example, cyt-PTPε is highly sensitive to ALN and N-(5-(phenoxymethyl)-1,3,4-thiadiazol-2-yl) acetamide derivatives, and both robustly suppress OC formation and function in vivo.^135,140^

PTP-oc (encoded by *Ptpro*, also known as PTPϕ) is expressed in OCs as an atypical RPTP due to the lack of a significant extracellular domain (Fig. 3).^141^ Mice overexpressing PTP-oc are osteoporotic due to increased c-Src activation and OC activity.^142,143^ Targeted deletion of PTP-oc in RAW264.7 cells impairs RANKL-induced osteoclastogenesis, though the mechanism remains unknown. Reducing PTP-oc expression in rabbit OCs weakens OC-mediated bone resorption, whereas PTP-oc overexpression in U937 human monocytic cells drives their differentiation into OC-like cells and increases their ability to resorb bone (Fig. 6).^144,145^ According to a mechanistic study, PTP-oc regulates c-Src and NFκB activation by dephosphorylating c-Src Tyr527 and decreasing IκBα levels, respectively (Fig. 7).^146^ Recently, PTP-oc was proposed to be bifunctional by dephosphorylating c-Src at its inhibitory site Tyr527 or activating site Tyr416. The unique bifunctional outcome depends on Tyr399 phosphorylation of PTP-oc as a molecular switch for substrate selection.^147^ PTP-oc also regulates immunoreceptor tyrosine-based activation motif (ITAM) and inhibition motif (ITIM)-mediated signaling pathways in OC through c-Src. PP2 (a selective inhibitor of SFKs) treatment reportedly attenuates OC activity by blocking PTP-oc-dependent phosphorylation of SYK Y525/526, β3-integrin Y759, and SHP1.^148^ Conversely, PTP-oc overexpression increases phosphorylation of VAV1 Y160, VAV3 Y173, PLCγ Y783, and JAK2 Y1007/1008 and activation of Rac, which are downstream mediators of ITAM/SYK signaling.^148,149^ Thus, PTP-oc plays a central role in coordinating OC development and function by modifying phosphorylation of key signaling molecules involved in the ITAM/SYK, β3-integrin, and ITIM/SHP1 signaling pathways (Fig. 7).^146,150^

PTP1B and T-cell PTP (TC-PTP) are classical NRPTPs, but they share similar functions with OST-PTP, a classical RPTP, in OCs. For this reason, we summarize these three PTPs in this section. OST-PTP and PTP1B regulate insulin-evoked signaling in OCs and OBs.^58^ In the latter, OST-PTP and PTP1B limit insulin signaling by dephosphorylating IR, which is required for forkhead box protein O1 and osteoprotegerin (OPG) expression. OB-produced OPG is a soluble decoy receptor for RANKL that competes with RANK to bind RANKL and mitigates RANK signaling and OC development and function.^58,151,152^ Zee et al. tested 37 mammalian classical PTPs for their ability to bind to endogenous IR in OBs and be upregulated by isoproterenol.^153^ The authors found that TC-PTP inhibits bone resorption by upregulating OPG expression by OBs, mimicking the effect of OST-PTP. Furthermore, TC-PTP deletion decreased bone mass and increased OC density.^154^ Taken together, OST-PTP, PTP1B, and TC-PTP are considered negative regulators in osteoclastogenesis by promoting OPG expression in OBs (Figs. 6, 7).

### Classical NRPTPs

Mouse models with spontaneous null (*me/me*) or hypomorphic (*me*^*v*^*/me*^*v*^) mutations in SHP1 exhibit reduced bone mass and cortical thickness. Further analysis of these mutants has revealed an increase in OC number and activity, indicating that SHP1 negatively regulates OC formation and function (Fig. 6).^74,75^ Consistent with these observations, overexpression of the dominant-negative SHP1 C453S mutant in RAW264.7 cells significantly enhanced OC formation and survival. This mutation also contributed to assembly of the RANK/TNF receptor-associated Factor 6 (TRAF6) signaling complex in response to RANKL^155^ to promote NFκB activation and PI3K p85 subunit phosphorylation, suggesting that SHP1 negatively modulates osteoclastogenesis by controlling the binding of TRAF6 to RANK (Fig. 7).^155^ Furthermore, macrophages from SHP1 mutant mice are hyperresponsive to M-CSF, indicating that SHP1 negatively regulates M-CSF signaling in OCs.^74,75^

By influencing inhibitory Ig-like receptor signaling, SHP1 has been implicated in osteoclastogenesis. Paired Ig-like receptor (PIR)-B, leukocyte Ig-like receptor B, and platelet endothelial cell adhesion molecule 1 are expressed in OC precursors. These receptors harbor ITIMs that activate SHP1 in the presence of RANKL and M-CSF and suppress OC development and bone resorptive activity in vitro (Fig. 7).^156,157^ SYK is crucial for bone mineral homeostasis through osteoclastogenesis modulation,^158^ which is mediated by SYK’s recruitment and regulation of ITAM-containing DAP12 or FcRγ.^159^ Importantly, SHP1 controls SYK activation in OCs by modifying its phosphorylation (Fig. 7)^148,160^ and inhibits ITAM-containing receptor signaling by directly blocking the binding and activation of PI3K.^161^ Ly49Q is another ITIM-bearing type II transmembrane protein that competes with PIR-B for binding to SHP1. Knockdown of Ly49Q results in a significant reduction in OCs in vitro, functioning as a positive regulator of osteoclastogenesis.^162^

DC-STAMP is a seven-transmembrane receptor-like protein containing an ITIM motif in its cytoplasmic tail that is known to be essential for cell-to-cell fusion during osteoclastogenesis.^163^ Upon phosphorylation, DC-STAMP physically interacts with SHP1 and CD16 to modulate RANKL and M-CSF-evoked signaling events and to promote cell fusion.^164^ HLA-G is an immunosuppressive molecule mainly expressed by osteoblastic cells. SHP1 is also required for HLA-G5-mediated inhibition of osteoclastogenesis due to its binding to ITIM-bearing HLA-G5 receptors Ig-like transcript 2 (ILT2) and ILT4 (Fig. 7).^165^ Inactivation of SHP1 by ROS-induced oxidation enhances OC viability (Fig. 7),^166^ and SHP1 can be recruited and activated by the ITIM of SIRPα to repress OC formation and function (Fig. 7).^167,168^ CD47 is a ligand for SIRPα.^169^ Stromal cells lacking CD47 or expressing SIRPα mutants lacking the cytoplasmic domain show a defect in osteogenic differentiation and supporting osteoclastogenesis, suggesting that CD47-evoked SIRPα-SHP1 signaling is critical for stromal cells to support osteoclastogenesis.^170^ Pao et al. generated a *Ptpn6*^*fl/fl*^ floxed allele;^171^ however, no apparent skeletal phenotype in monocyte/macrophage-specific SHP1-deficient mice (*Ptpn6*^*fl/fl*^*;LysM-Cre or Ptpn6*^*fl/fl*^*;F4/80-Cre*) was found, suggesting that SHP1 is dispensable for the proliferation and differentiation of OC precursors and mature OCs.^172^

In the late 1990s, SHP2 was implicated in M-CSFR signaling and possibly OC development.^173^ Using myeloid progenitor 32D cells that express M-CSFR, Lee reported that SHP2 and GAB2 were tyrosyl phosphorylated and associated with each other in response to M-CSF stimulation.^173^ These observations were confirmed in FDC-P1 cells expressing exogenous M-CSFR, which phosphorylated the scaffold adaptor GAB2 and recruited SHP2, leading to ERK activation and osteoclastic differentiation.^174^ To study SHP2’s role in osteoclastogenesis and OC functional regulation, *Ptpn11* floxed alleles were crossed into inducible or OC lineage-specific Cre mouse lines. Using *Cmv-CreERT2* as a driver, Bauler et al.^175^ showed that mice with SHP2 deletion in multiple tissues caused early lethality, reminiscent of the phenotype of mice lacking SHP2 in hematopoietic cells.^176^ Affected SHP2 mutants were claimed to have defects in OC formation in vivo and in vitro, with increased bone mass, and the authors ascribed the OC and skeletal phenotypes to impaired M-CSF-evoked ERK and AKT activation and myeloid cell viability (Figs. 6, 7).^175^ Nevertheless, independent studies showed that SHP2 deletion in BMMs impairs ERK but enhances AKT activation after M-CSF stimulation via an ERK/RSK negative feedback signaling loop, leading to compromised cell proliferation without an apparent effect on cell survival (Fig. 7).^177^ In the abovementioned studies, two different *Ptpn11* floxed alleles and Cre lines were used, and additional investigations are required to clarify the cause of the discrepancy.

Further mechanistic studies have revealed that SHP2 regulates osteoclastogenesis by promoting the fusion of preosteoclasts. This action is mediated through the RANKL/NFATc1 signaling axis, as SHP2 deficiency markedly reduces expression of NFATc1, a master TF that is indispensable for OC terminal differentiation (Fig. 7).^178,179^ SHP2 also participates in the IL6- or IL11-induced gp130/Ras/ERK signaling pathway that promotes osteoclastogenesis (Fig. 7).^180–182^ gp130-dependent cytokines bind to target α-receptor subunits that form a receptor complex containing the gp130 coreceptor subunit. This complex activates JAK/STATs and SHP2/Ras/ERK signaling pathways, enabling ligand- and tissue-specific activation of distinct sets of target genes and biological responses. Characterization of mice bearing gp130^*Y757F/ Y757F*^ mutations has demonstrated that SHP2 is required for the gp130-induced RAS/ERK activation accounting for osteoclastogenesis inhibition (Fig. 7).^182,183^ Furthermore, SHP2 modifies osteoclastogenesis by controlling secretion of RANKL by OBs and osteocytes. Mice lacking SHP2 in OBs display enhanced osteoclastogenesis.^38^

Both SHP1 and SHP2 have been purported to regulate c-Src activity, which is essential for OC function. Mice lacking c-Src form multinucleated OCs but develop osteopetrosis due to defects in ruffled border formation and bone resorption.^184,185^ Mechanistically, SHP1 and SHP2 regulate c-Src activation by promoting c-Src Y416 phosphorylation^77^ or by dephosphorylating the inhibitory Y527 of c-Src.^186^

PTP-PEST regulates OC differentiation and function by influencing RANKL-evoked fusion of preosteoclasts and their polarization, migration, and spreading.^187^ PTP-PEST also regulates OC adhesion and function by modifying podosome formation and motility.^99,138^ Under physiological conditions, PTP-PEST localizes to the podosome peripheral sealing zone of resorbing OCs.^138,188–190^ PTP-PEST overexpression activates c-Src and subsequently ARP2 and WASP to increase OC sealing ring formation and bone-resorbing activity (Fig. 7).^191^ Therefore, PTP-PEST is a critical component of the OC podosome signaling complex. PYK2 is highly expressed in OCs and is another substrate of PTP-PEST. PYK2 is activated by phosphorylation of Y402, which is dephosphorylated by the coordinated action of PTP-PEST and dynamin. PYK2 dephosphorylation by PTP-PEST causes impaired OC function and increased bone mass (Fig. 7).^192^ Taken together, PTP-PEST positively regulates OC differentiation, adhesion, and function.

### DUSPs

MKP1 is essential for osteoclastogenesis and OC functional regulation. Valerio et al. reported that MKP1 regulates OC formation through the RANKL/NFATc1 axis.^193,194^ OC activity is augmented by MAPK (p38 and JNK) pathway activation but negatively regulated by MKP1 in vitro (Fig. 7).^195,196^ Consistent with these findings, compared with *Mkp1*^*+/+*^ controls, *Mkp1*^*−/−*^ mice show elevated OC resorptive activity.^196^ Importantly, MKP1 deficiency increases OC formation and activation in response to TNF in vitro and causes extensive bone loss and arthritis in vivo.^197^ Moreover, MKP1 inhibits alveolar bone loss resulting from elevated OC formation induced by bacterial pathogens,^195^ likely by dephosphorylating and inactivating all three groups of MAPKs (p38, JNK, and ERK) after lipopolysaccharide stimulation.^198^ In addition to directly regulating OC formation and function, MKP1 and DUSP5 restrain osteoclastogenesis and OC activation by inhibiting expression and secretion of RANKL and CXCL10 by OBs (Fig. 7).^199–201^ Thus, MKP1 plays a role in protecting against inflammatory bone loss and may serve as a therapeutic target.

Traditionally, OCs are thought to undergo apoptosis after completing bone resorption.^202^ This concept, however, has been changing since discoveries showing that multinucleated OCs can split into multiple daughter cells, called osteomorphs, and that osteomorphs can refuse and form giant OCs in response to RANKL (Fig. 6).^203^ scRNA-seq has indicated that osteomorphs are transcriptionally distinct from OCs and macrophages based on expression of atypical OC genes, including *Dusp2* and *Ptp4a3* (encoding PRL3), suggesting that DUSP2 and PRL3 might be involved in OC fission and osteomorph recycling (Fig. 6). These concepts are worthy of further investigation.

The DUSP PTEN plays a role in osteoclastogenesis. Mice lacking PTEN have an increased number of OCs in vivo compared with wild-type controls due to elevated NFATc1 expression (Fig. 7). Nevertheless, affected PTEN mutants do not exhibit an osteopenic phenotype because of compensatory OB hyperactivity.^204^ PTEN overexpression in RAW264.7 cells leads to increased apoptosis and decreased OC differentiation as a result of compromised AKT, BAD, and IκBα phosphorylation and NFκB expression (Fig. 6).^205^ PTEN also inhibits OC migration induced by osteopontin by inactivating AKT.^205^ AKT phosphorylates and inhibits GSK3β, which activates PTEN via phosphorylation at Thr366, leading to suppression of RANKL-induced osteoclastogenesis (Fig. 7).^206^ PTEN is also a molecular target of miR-214 and miR-142-5p, which downregulate PTEN expression to promote OC differentiation and activation.^207,208^ Collectively, PTEN is a key regulator in RANKL-induced osteoclastogenesis, likely by modifying phosphorylation and activation of AKT and GSK3β.

In summary, 14 PTPs are reported to modulate osteoclastogenesis and OC function. Among these PTPs, PTP-OC, SHP2, CD45, PTP-PEST, and cyt-PTPε seem to positively influence these processes, whereas OST-PTP, PTP1B, TC-PTP, MKP1, DUSP5, DUSP2, SHP1, and PTEN act in the opposite manner. Furthermore, OST-PTP, PTP1B, TC-PTP, MKP1, and DUSP5 modify osteoclastogenesis indirectly by influencing OB secretion of multiple cytokines, such as OPG, RANKL, and CXCL10. DUSP2 and PRL3 were recently implicated in OC fission; how they function at the molecular level remains unclear and warrants further study.^203^ Conceivably, a single PTP can modify one or multiple signaling pathways, and multiple PTPs can target a single signaling molecule or pathway in OCs, reflecting the complexity of the regulatory networks during osteoclastogenesis. Given the crucial role of OC in skeletal development, remodeling, and bone mineral homeostasis, a few drugs that target OC formation and function to mitigate bone resorption have been developed, such as bisphosphonates, calcitonin, and denosumab. However, long-term administration of these drugs can cause adverse effects, such as osteonecrosis of the jaw, atypical femoral fracture, and hypocalcemia.^209^ Thus, searching for novel medications by targeting PTPs and their associated signaling pathways in OCs is an important future direction.

## PTPs in chondrocyte development and cartilage homeostasis

Chondrogenesis is a dynamic process that involves recruitment, migration, and condensation of MSCs, followed by differentiation into OCPs and proliferating and hypertrophic chondrocytes.^210^ Chondrocytes are the only native cells in cartilaginous connective tissue responsible for producing and maintaining the cartilage matrix. Here, we review 13 PTPs known to modulate chondrogenesis and cartilage homeostasis.

### Classical RPTP

RPTPζ and its ligand pleiotrophin, a heparin-binding growth factor, are reported to be elevated in both the cartilage and subchondral bone of osteoarthritis (OA) patients^211,212^ and in the intervertebral disc and endplate chondrocytes of mice with spinal deformities caused by static axial and asymmetrical mechanical loading.^213^ Although both RPTP ζ and pleiotrophin play a vital role in OA pathophysiology and intervertebral disc homeostasis, the mechanism underlying these processes remains incompletely understood.

OST-PTP and the gamma isoform of PTPPBS (PTPPBSγ) are expressed in the mesenchyme of craniofacial bones, ribs, limbs, and Meckel’s cartilage. During initial chondrogenesis, OST-PTP and PTPPBSγ mRNA expression exclusively localizes to the perichondrium of all endochondral elements.^56,214^ PTPPBSγ has been demonstrated to regulate the proliferation of chondroblasts at an early stage,^214^ but the function of OST-PTP in the perichondrium is still unknown. As inhibiting PTPPBSγ increases the proliferation of chondroblasts and the population of mature chondrocytes,^56,214^ PTPPBSγ negatively regulates chondroblast proliferation and maturation (Fig. 4).

### Classical NRPTP

**Fig. 8:**
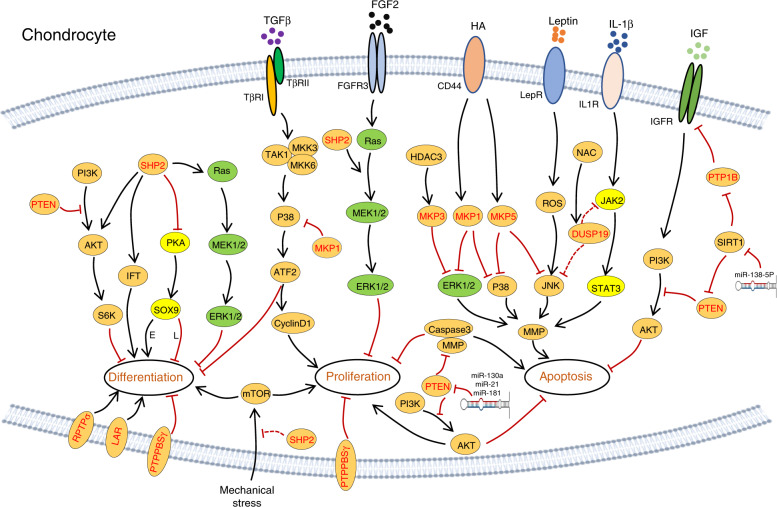
Schematic diagrams illustrating signaling pathways and PTPs involved in chondrogenesis. Known signaling pathways that modulate the survival, proliferation, differentiation, and function of chondrocytes are connected by lines with an arrow or “┴” indicating a promoting or inhibiting regulatory role, respectively. In all signaling pathways, PTPs are marked in red. SOX9 is a crucial transcription factor for the differentiation of chondrocytes. Elevated SOX9 in the growth plate promotes early chondrocyte differentiation (“E” was labeled next to the line) but halts terminal differentiation and osteogenic transdifferentiation (“L” is labeled next to the line)

PTP1B plays a role in cartilage homeostasis. It is reported that PTP1B dephosphorylates IGFR and impairs activation of its downstream effectors AKT and MDM2, promoting chondrocyte apoptosis. This apoptotic effect is suppressed by sirtuin 1 (SIRT1),^215^ a member of the SIRTUIN family nucleic proteins with deacetylase activity, by repressing PTP1B expression.^216^ The antiapoptotic effect of SIRT1 is further supported by the observation of elevated chondrocyte apoptotic death in *SIRT1*^*−/*^^−^ mice.^217^ Collectively, these data indicate that PTP1B may be a druggable target for cartilage anti-degeneration (Figs. 4, 8).

SHP2 is a key regulator of chondrogenesis and cartilage homeostasis. To study SHP2’s role in cartilage, chondrocyte developmental stage-specific SHP2-deficient mice were generated using the “Cre-LoxP” system. Phenotypic characterization of these SHP2 mutants demonstrated that SHP2 negatively regulates chondrogenic differentiation of *Prrx1*^*+*^ OCPs (Fig. 4).^36,37^ SHP2 deletion in *Col2a1*^*+*^ cells causes dwarfism, exostoses, enchondromas, and low BMD in mice; its deletion in *Col10a1*^*+*^ hypertrophic chondrocytes results in a mild phenotype with an elongated layer of hypertrophic chondrocytes and a slight decrease in BMD.^23,218^ Further mechanistic studies have revealed that SHP2 negatively regulates the proliferation of growth plate chondrocytes and is required for terminal differentiation and osteogenic transdifferentiation (Fig. 4).^23^ These findings are corroborated by RNA-seq studies of chondrocyte maturation. SHP2 deletion increases the transcript abundance of genes associated with early chondrogenesis but decreases that of those associated with terminal differentiation.^219^ At the molecular level, SHP2 deletion in *Prrx1*^*+*^ cells compromises ERK and AKT activation^37^ but enhances PKA activation and the phosphorylation, sumoylation, and transcriptional activity of SOX9 (Fig. 8).^36^ Elevated SOX9 in growth plate hypertrophic chondrocytes halts their terminal differentiation and osteogenic transdifferentiation.^23^ Conversely, heterozygous *Ptpn11*^*D61G/+*^ mice display elevated activation of the Ras/ERK pathway and consequently reduced chondrocyte proliferation and a shorter growth plate. U0126 treatment partially reverses growth plate abnormalities by enhancing chondrocyte differentiation.^220^ Importantly, SHP2 negatively regulates chondrocyte proliferation by promoting FGF2-evoked Ras/ERK signaling, in which transient ERK activation appears to be crucial for mitogenic signaling; however, sustained activity frequently leads to growth arrest (Fig. 8).^221^

SHP2 has also been shown to regulate chondrocyte differentiation by modifying vinculin and mTOR signaling.^222,223^ Vinculin is a cytoskeletal protein that coordinates cell adhesion and/or signaling between the extracellular milieu and the cell via integrins and cadherins. Vinculin knockdown enhanced SHP2 phosphorylation but impaired downstream ERK1/2 activation in chondrocytes, indicating that vinculin indirectly regulates SHP2 activity in chondrocytes.^222^ By inhibiting mTOR activation, SHP2 also negatively regulates mechanically induced chondrocyte proliferation and differentiation.^223^

In addition to long bone cartilage, SHP2 regulates orofacial cartilage development. Kamiya et al. reported that SHP2 deficiency in orofacial cartilage chondrocytes causes severe mandibular condyle deformity due to expanded cartilage in the trabecular area, along with a reduction in the number and length of cilia.^224^ Mechanistically, SHP2 may regulate ciliogenesis and cilia-mediated mechanotransduction in chondrocytes by positively influencing intraflagellar transport components (Fig. 8).^224^ The detailed molecular mechanism, however, remains elusive.

### DUSPs

Activation of MKP1 after binding of hyaluronic acid to CD44 suppresses ERK1/2 activation and MMP production in human chondrocytes.^225^ Additionally, activated MKP1 dephosphorylates p38 to promote chondrocyte maturation while inhibiting chondrocyte proliferation through the TGF-β/TAK1/ATF-2 axis, suggesting that modulating p38 activation has potential clinical therapeutic value (Figs. 4, 8).^226,227^ MKP3 and MKP5 (encoded by *Dusp6* and *Dusp10*, respectively) negatively regulate chondrocyte terminal differentiation and apoptosis by dephosphorylating MAPK and reducing MMP13 expression in chondrocytes.^228,229^ Similar to MKP1, MKP5 is induced by hyaluronan-CD44 interaction, which reduces MMP13 expression by dephosphorylating p38 MAPK and JNK.^229^ Gene expression microarray analysis indicates that the abundance of both MKP5 and DUSP10 is decreased in OA cartilage (Figs. 4, 8).^230^

DUSP19 (encoded by *Dusp19*)^231^ inhibits chondrocyte apoptosis by inactivating JNK activation. DUSP19 levels are decreased in human OA cartilage and correlate negatively with leptin in a rat OA model; ectopic expression of DUSP19 alleviates leptin-induced chondrocyte apoptosis.^232^ Nonetheless, these observations conflict with Yao’s finding that DUSP19 inhibits IL1β-induced chondrocyte apoptosis and MMP expression^233^ as a result of reduced JAK2 and STAT3 activation, though it remains unknown how DUSP19 modifies JAK2 and STAT3 activation (Figs. 4, 8).^233^ Further studies are required for clarification.

PTEN is indispensable for cartilage development and homeostasis. Mice lacking PTEN in *Col2α1*^*+*^ cells show skeletal abnormalities manifesting as disorganized growth plates, matrix overproduction, and skeletal overgrowth.^116,234,235^ PTEN-deficient chondrocytes exhibit delayed and asynchronous differentiation due to enhanced signaling along the PI3K-AKT-S6 axis and enhanced ER stress (Figs. 4, 8);^116,234^ similar findings were reported for PTEN-knockdown chondrocytes,^236^ suggesting that PTEN is essential for chondrocyte stress adaptation. PTEN also participates in miR-130a-induced chondrocyte proliferation and OA alleviation.^237^ Circulating miR-130a is decreased in OA patients compared to healthy controls. Downregulation of miR-130a reduces chondrocyte proliferation and increases chondrocyte apoptosis, and this action may occur through PTEN/PI3K/AKT signaling suppression. Regardless, the coherent reduction in PI3K in chondrocytes expressing anti-miR-130 suggests that this regulation is PTEN specific; further studies are needed.^237^

Consistent with the above, PTEN levels were found to increase significantly in OA cartilage,^238^ and PTEN knockdown was able to improve cartilage matrix synthesis and chondrocyte proliferation.^236,238^ MicroRNAs that modify PTEN expression also regulate intervertebral disc (IVD) homeostasis. PTEN downregulation by miR-21 or miR-138-5p alleviates IVD degeneration by decreasing nucleus pulpous (NP) cell apoptosis, a mechanism mediated through PI3K/PTEN/AKT signaling pathway activation (Fig. 8).^239,240^ In contrast, it has been reported that PTEN downregulation by miR-21 accelerates IVD degradation by promoting MMP production and inhibiting NP-cell autophagy^241^ and that miR-181 inhibits chondrocyte proliferation and drives chondrocyte apoptosis by increasing expression of caspase-3, PARP, MMP2, and MMP9 (Fig. 8).^242^ Taken together, PTEN is crucial for regulating the proliferation and survival of chondrocytes, though the outcome of PTEN activation or downregulation by microRNAs in cartilage remains controversial.

PRL1, encoded by *Ptp4a1*, is a member of the DUSP family. PRL1 expression varies depending on the chondrocyte developmental stage in mice.^243^ It is highly expressed in condensing prechondrogenic cells of the vertebrae at E13.5, whereas *Prl1* mRNA abundance drops in hypertrophic chondrocytes at E18.5, suggesting a functional role for PRL1 in chondrocyte differentiation.^243^

In summary, studying mouse models that carry PTP GOF and loss-of-function (LOF) mutations in chondroid cells has uncovered a crucial role for PTPs in chondrogenesis. Indeed, they modify almost every aspect of cartilage biology, including the fate determination of stem cells, chondroid cell proliferation, hypertrophic differentiation, and osteogenic transdifferentiation. Most importantly, somatic LOF mutation in SHP2 causes metachondromatosis and benign cartilage syndrome in both humans and mice, suggesting that PTPs are pivotal for cartilage homeostasis. Therefore, identifying the substrate(s) of individual PTPs and understanding how they function in chondroid cells will provide insight into developing novel therapeutics to combat cartilaginous diseases.

## PTPs in skeletal diseases

PTPs not only physiologically regulate bone and cartilage development but also participate in the pathogenesis of several human skeletal disorders. Some PTP mutations involved in the pathogenesis of human skeletal diseases have already been discussed in this review; here, we focus only on those remaining.

### Syndromes with skeletal abnormalities

Genetic mutations in several PTPs can cause syndromes with skeletal manifestations in humans, e.g., NS, Noonan-like/multiple giant cell lesion syndromes (NS/MGCLS), Leopard syndrome (LS), and Otofaciocervical syndrome (OS). NS, NS/MGCLS, and LS are rare diseases partially caused by SHP2 mutation, accounting for ~50% of NS cases; some of them also have multiple giant cell lesions,^83,244,245^ and SHP2 missense mutations account for ~80% of LS cases.^246^ NS, NS/MGCLS, and LS result in skeletal abnormalities, including dysmorphic facies, short stature, spinal deformities, pectus excavatum or carinatum, and abnormal elbow articulation, in addition to impacting other organs.^247,248^ These skeletal abnormalities can be partially explained by dysregulation of Ras/MAPK signaling caused by SHP2 mutations in germline cells and abnormal endocrine homeostasis and skeletal cell differentiation.^247,249,250^

OS is an autosomal dominant disorder caused by mutation in the gene encoding EYA1, an Asp-based phosphatase that functions as a coactivator of the homeodomain TF SIX1.^14^ OS clinically manifests as hearing loss, branchial fistulae, low-set ears, facial abnormalities, mental retardation, vertebral defects, low-set clavicles, winged scapulae, and sloping shoulders.^251^ SOX9 ChIP-seq shows that *Eya1* is a SOX9 target in chondrocytes,^252^ which might partially explain the skeletal phenotypes of EYA1 mutants. In addition, Zhang et al. proposed that EYA1 dephosphorylates pThr2122 of NOTCH1, consequently increasing its stability and activity in the epibranchial placode cells that play a critical role in craniofacial morphogenesis.^253^ Thus, EYA1 mutations may cause craniofacial abnormalities by modifying the NOTCH signaling pathway.

### Bone tumors

RPTPδ mutations are associated with osteosarcoma and Ewing sarcoma. Such mutations occur in up to 37.5% of patients with metastatic Ewing sarcoma.^254–256^ PTPL1 (encoded by *Ptpn13*) is a direct transcriptional target of EWS-FLI1 that modulates EWS tumorigenesis.^257^ RPTPζ is also involved in the pathology of osteosarcoma. In one study, the level of RPTPζ transcripts was higher in 73% (22/30) and lower in 27% (8/30) of osteosarcoma samples from patients than in five healthy controls.^258^ There was no correlation between RPTPζ expression, clinicopathological parameters, or survival rate. However, RPTPζ deficiency promoted osteosarcoma development in Trp53-heterozygous mice.^47^ Loss of PTEN has also been implicated in bone malignancies, including osteosarcoma.^259^ PTEN not only inhibits the proliferation, migration, and invasion of osteosarcoma cells but also facilitates their apoptosis.^259^ Thus, PTEN is an important tumor suppressor in skeletal tissues.

### Cartilage tumors

Multiple cartilaginous bone tumors are characteristic of cartilage tumor syndromes, often causing significant morbidity and predisposing patients toward chondrosarcoma. In general, the etiology of cartilage tumors remains elusive. However, linkage analysis using high-density SNP arrays and whole-genome sequencing has revealed that *PTPN11* LOF mutation causes MC.^260,261^ Analyzing the skeletal phenotype of mice lacking SHP2 in cathepsin K (*Ctsk*^*+*^)-expressing cells showed that SHP2 functions as a tumor suppressor in cartilage, negatively regulating the proliferation and chondrogenic differentiation of chondroprogenitors.^13^ Although CTSK is traditionally considered a marker of OCs, this view rapidly changed since the discovery of Ctsk promoter activity in cells within the groove of Ranvier and cartilage tumors.^13^ Furthermore, CTSK has recently been reported to be expressed in progenitors of the periosteum and tendon tissue.^262,263^ SHP2 deletion in *Ctsk*^*+*^, *Prrx1*^*+*^, and *Col2a1*^*+*^ chondroprogenitors results in enchondromas and osteochondromas in mice, which phenotypically mimic human MC.^13,23,36,264^ Other studies have revealed that over 50% of MC cases in humans involve frameshift, nonsense, splice-site mutations, or deletions in *Ptpn11.*^13,260,264^ Interestingly, mice lacking SHP2 in CD4^+^ cells develop cartilaginous tumors,^265^ and their skeletal phenotypes were recapitulated in mice with SOS and ERK deletion in CD4^+^ cells.^266,267^ Together, these data suggest that canonical RAS/SHP2/ERK signaling is pivotal for cartilage homeostasis^219^ and that the CD4 promoter is transiently activated in subset chondroid cells during development; and the biology of SHP2 in cartilage cells remains incompletely understood, warranting further investigation.

## Conclusions

Protein tyrosyl phosphorylation is an important cellular regulatory mechanism in the skeletal system, as many enzymes and signaling pathways are activated or deactivated by this posttranslational protein modification through the action of PTKs and PTPs. Given that the signaling networks involving PTKs are highly complex, the PTPs involved also play an intricate role. In this review, we discuss the mechanism of action of 28 PTPs, with particular attention to their roles in bone, cartilage, and OCs under physiological and pathological conditions. Several classes of RPTPs, NRPTPs, DUSPs, and LMW-PTP are found in multiple complex signaling networks in OBs, OCs, and chondrocytes. Each PTP may have distinct substrates depending on the type of skeletal cells and their developmental stage; or it may target the same substrate but with distinct biological outcomes, exhibiting cellular context-specific or stage-specific effects. In light of these observations, it becomes clear why the same PTP mutation causes various skeletal manifestations.

Over the past decade, phosphatase targeting has moved to the forefront of drug development. Several PTPs are attractive pharmacological targets, including cyt-PTPε for osteoporosis,^26,137^ SHP2 for cartilage regeneration,^36^ and RPTPζ for IVD anti-degeneration.^213^ Despite promising progress in developing chemically allosteric inhibitors,^268–270^ finding specific, cell-permeable, and clinically effective compounds for PTPs, in contrast to PTKs, remains challenging due to the cellular context-specific effects and the lack of cell permeability and selectivity across members of the PTP family. An exciting new approach for PTP targeting is the design of PROTACs to induce PTP degradation.^271^ Targeting chimera PROTACs are bifunctional compounds that bind to a target protein and to an E3 ubiquitin ligase, causing the target protein to be ubiquitinated and degraded. PROTACs have been successfully used to degrade SHP2 in various types of cells,^272^ including bone and cartilage cells (LW and WY, unpublished data), with high specificity and cell permeability. Considering the spatial structure of skeletal tissue (e.g., articular cavity and IVD) and cutting-edge nanodelivery technology, local administration of PTP inhibitors or degraders may become possible for therapeutic application without apparent adverse effects.

Although the number and function of PTPs in skeletal tissues remain unclear, the most crucial questions are regarding physiological substrates of these PTPs and the specific signaling pathways they regulate in skeletal cells. Of course, such questions are complex. For example, PTP1B was the first PTP discovered 20 years ago, and its substrates in various tissues remain incompletely understood.^273^ Nevertheless, advanced methodologies, such as lineage- and stage-specific gene knockout mouse models, ‘substrate trapping’ PTP mutants, and phosphoproteomics, will be valuable in addressing these questions. Overall, a systematic assessment of the consequences of PTP chemical inhibition and degradation will be crucial to this endeavor and in understanding bone physiology and skeletal disorders in the future.
